# Analytical Solution of Mixed Electroosmotic/Pressure Driven Flow of Viscoelastic Fluids between a Parallel Flat Plates Micro-Channel: The Maxwell Model Using the Oldroyd and Jaumann Time Derivatives

**DOI:** 10.3390/mi11110986

**Published:** 2020-10-31

**Authors:** Laura Casas, José A. Ortega, Aldo Gómez, Juan Escandón, René O. Vargas

**Affiliations:** 1 SEPI-ESIME Zacatenco, Departamento de Diseño, Instituto Politécnico Nacional, Av. Luis Enrique Erro S/N, Unidad Profesional Adolfo López Mateos, Zacatenco, Alcaldía Gustavo A. Madero, Ciudad de México, 07738, Mexico; lacasas3@yahoo.com.mx (L.C.); jaortega@ipn.mx (J.A.O.); 2 Departamento de Ingeniería, Universidad Nacional Autónoma de México, FES Cuautitlán, Sección Mecánica, Av. Teoloyucan Km 2.5, Col. San Sebastián Xhala, Cuautitlán Izcalli 54714, Estado de México, Mexico; aldo009.gl@gmail.com; 3 Departamento de Termofluidos, Instituto Politécnico Nacional, SEPI-ESIME Azcapotzalco, Av. de las Granjas No. 682, Col. Santa Catarina, Alcaldía Azcapotzalco, Ciudad de México 02250, Mexico; jescandon@ipn.mx

**Keywords:** electroosmotic, pressure driven flow, viscoelastic fluid, oldroyd derivate, jaumann derivate, microchannel

## Abstract

In the present work, an analytical approximate solution of mixed electroosmotic/pressure driven flow of viscoelastic fluids between a parallel plates microchannel is reported. Inserting the Oldroyd, Jaumann, or both time derivatives into the Maxwell model, important differences in the velocity profiles were found. The presence of the shear and normal stresses is only close to the wall. This model can be used as a tool to understand the flow behavior of low viscosity fluids, as most of them experiment on translation, deformation and rotation of the flow. For practical applications, the volumetric flow rate can be controlled with two parameters, namely the gradient pressure and the electrokinetic parameter, once the fluid has been rheologically characterized.

## 1. Introduction

Microfluidics deals with the behavior, precise control and manipulation of fluids that are geometrically constrained to a small, typically sub-millimeter scale. It is a multidisciplinary field intersecting engineering, physics, chemistry, microtechnology and biotechnology [1]. In this regard, microfluidic devices, microscale laboratories on a microchip (lab-on-a-chip, LOC) and micro-electro-mechanical-systems (MEMS) have become important due to their wide applications and the rapid development of the micro- and nano-technology. Important applications are found in thermo/mechanical (heating/cooling systems, pumping, design, control, saving material and energy), chemical (mixing, separation and homogeneous/heterogeneous reactions), biomedical (collection, dispensing, detection, mixing and species separation) and pharmaceutical (drug delivery) industries. In this context, the role of electrokinetic phenomena in the tasks of microfluidic devices has grown over the years; the first experimental and theoretical developments on electrokinetic transport phenomena are attributed to Reuss (1809), Helmholtz (1879) and Smoluchowski (1903) [2]. The four key electrokinetic phenomena are streaming potential, electroosmotic flow, electrophoresis and sedimentation of charged suspensions (for details, see [2,3,4,5]). The electrokinetic transport combines two driving mechanisms, electrophoresis and electro-osmosis; the first one refers to the motion of a charged particles in a fluid under the influence of an electric field, and the second one refers to the movement of a volume of an aqueous solution adjacent to a solid charged surface when an external electric field is applied tangentially along the surface [1,2,4,5]. Therefore, it is fundamental to understand the electrokinetic phenomena involved in these microfluidic devices [1,6].

In recent decades, scientific researchers from all areas have focused on the important aspects of microfluidics such as the hydrodynamic behavior of electroosmotic flows of Newtonian [7,8,9,10,11] and non-Newtonian fluids [12,13,14,15,16,17], different geometries [18,19,20,21,22,23,24,25,26,27,28,29], high/low zeta potentials [17,30,31,32,33,34,35], the Joule heating effect [7,14,29,30,36] and numerical simulations [7,17,18,31,37].

Many of these studies assume Newtonian fluids with constant viscosity; however, in microfluidics devices, the substances to be analyzed are usually non-Newtonian such as biofluids (saliva, blood, proteins and DNA), colloidal suspensions or polymers [6,17]. To characterize the hydrodynamics, it requires starting from the Cauchy momentum equation instead of the Navier–Stokes equation, together with a constitutive equation that describes the dependence of the viscosity with the strain rate. The constitutive equations used in electroosmotic flow (EOF) can be grouped into three categories: inelastic, viscoelastic and kinetic models [17,38,39,40]. Inelastic models include the power law, Herschel–Bulkley, and Bingham. Viscoelastic models include the upper-convected Maxwell (UCM), Oldroyd-B, Carreau, second-grade fluid, third-grade fluid, Burgers and Phan–Thien–Tanner (PTT). The kinetic models include the finitely extensible dumbbells with a Peterlin approximation of the spring force (FENE-P). An exhaustive review of the non-Newtonian effects on electrokinetics carried out until 2013 is summarized in [17]. About the viscoelastic models, an analytical solution for one-dimensional electroosmotic flow between oscillating micro-parallel plates of viscoelastic fluids represented by a single-mode generalized Maxwell model, used to understand the flow characteristics, was presented by Liu et al. [41]. Analytical solutions for the oscillatory shear flow using the multi-mode upper-convected Maxwell model driven by electroosmotic forcing with asymmetric wall zeta potentials, to investigate the influence of the relevant dimensionless parameters on the normalized velocity profiles when imposing an externally potential field, was presented by Sadek and Pinho [28]. The transient electroosmotic flow through with the Maxwell fluid model in a slit microchannel with asymmetric zeta potentials was studied by Escandón et al. [42]. The transient electroosmotic flows of Maxwell fluids in a micro-parallel channel and a microtube was presented by Li et al. [43]. In addition, the transient electroosmotic flow of generalized Maxwell fluids with fractional derivative in a straight pipe with circular cross-section was investigated by Wang et al. [44]. On the other hand, results concerning the transient electroosmotic flow driven by AC electric fields were obtained by Jian et al. [45] and Liu et al. [41], who obtained an analytical solution of the time-periodic electroosmotic flow for the generalized Maxwell fluids through a rectangular microchannel under the Debye–Hückel approximation. The start-up from the rest of the electroosmotic flow of Maxwell fluids in a rectangular microchannel with asymmetric high zeta potentials at the walls was studied by Jiménez et al. [46]. An analytical solution of the unsteady electroosmotic flow of Oldroyd-B fluid in a capillary analyzing the relaxation and retardation times was presented by Zhao et al. [47]. The flow pattern of electroosmotic flow in a porous microchannel with a second-grade viscoelastic fluid under alternating electric field was studied by Misra and Chandra [34]. Analytical and numerical solutions of an electroosmotic flow with a third-grade fluid between micro-parallel plates, to analyze the influence of the principal parameters on the velocity profile, were presented by Akgul and Pakdemirli [37]. The steady-state of a conjugate heat transfer process in an electroosmotic and fully developed laminar flow with a Phan–Thien–Tanner fluid, including Joule heating effects, was solved numerically and asymptotically by Escandón et al. [26]. A non-linear solution of a viscoelastic fluid under the combined influence of electrokinetic and pressure forces using the Debye–Hückel approximation was properly coupled by Afonso et al. [23]. A simple method to find the volumetric flow rate for various viscoelastic (UCM and PTT) electroosmotic flows through microchannels was presented by Park and Lee [24]. The numerical solution of the thermal transport of the steady electroosmotic flow in a slit microchannel, which considers constant wall heat fluxes, Joule heating, conjugate heat transfer and temperature-dependent properties, was investigated by Sadeghi et al. [48]. The steady electroosmotic flow considered asymmetric wall zeta potentials for the simplified PTT and FENE-P models was studied by Afonso et al. [49]; this work was extended by Dhinakaran et al. [25], inserting the full Gordon–Schowalter convective derivative in the PTT to analyze the critical shear rate and Deborah number for the onset of fluid instabilities. The two-dimensional numerical simulations for electrokinetic flow through a microchannel using the Carreau model was performed by Zimmerman et al. [50].

As seen in the previous paragraph, the viscoelastic models have been widely used to predict the electroosmotic flow behavior of fluids with non-Newtonian viscosity and elastic stresses. In this context and to extend the different physical interpretations of this kind of fluids flow, it is important to highlight that, in some of these models, the incorporation of the time derivative of the stress tensor can be done in two ways. One form is called the Oldroyd contravariant derivative (also called the codeformational derivative), which gives their components, in fixed coordinates, of the time derivative as observed in a coordinate system which translates and deforms with the flow field. Another time derivative is the Jaumann derivative (also called corotational derivative), which gives their components, in fixed coordinates, of the time derivative as observed in a coordinate system which translates and rotates with the local rotation [38,40]. The former is one of the most used in the electroosmotic flows [23,28,47,49,51].

In addition to the vast number of theoretical studies on electroosmotic flows presented above, the scientific community has carried out experimental investigations with non-Newtonian fluids. Song et al. [52] presented the first experimental study of the electrokinetic instability in viscoelastic fluid flow with conductivity gradients for mixing applications. Ko et al. [53] performed an experimental study of fluid elasticity and shear thinning on the electroosmotic flow of four types of polymer solutions in insulator-based dielectrophoresis, i.e., in a constriction microchannel, as an emerging technique to manipulate a variety of particles. In another work, Mukherjee et al. [54] investigated the electroosmotic flow of two immiscible viscoelastic fluids in a parallel flat plates microchannel, in their analysis the effect of a depletion layer is incorporated near the walls. To assess the theoretical predictions, the authors performed experiments on electro-osmosis using aqueous solutions of polyacrylamide. Their analysis reveals that neglecting the existence of a depletion layer results in grossly incorrect predictions of the transport of complex fluids. Pimenta and Alves [55] investigated the electroosmotic flow of viscoelastic fluids in cross-slot and flow-focusing microdevices, with especial focus on the onset of elastic instabilities. The experimental and numerical results suggest that the large stresses developed inside the electric double layer, together with the streamline curvature around the geometry corners, play a fundamental role in the onset and the dynamics of the observed electro-elastic instabilities. Bello et al. [56] and Olivares et al. [57] carried out a theoretical and experimental investigation for the electroosmotic mobility of polymeric solutions of methyl cellulose and carboxymethyl cellulose, respectively, with the behavior of a power law fluids. For their part, Huang et al. [58] conducted experiments on the electroosmotic driven flow using current monitoring and microscopy fluorescence methods and developed a theoretical model by coupling a generalized Smoluchowski approach with the power law constitutive model. Here, the fluid sample used in the experiment is a polyethylene oxide aqueous solution with several concentrations. The results show an enhancement of the electroosmotic velocity due to the shear thinning effect. Many other experimental works about electrokinetics of non-Newtonian fluids are included in the comprehensive review reported by Zhao and Yang [17], as well as in the dissertation carried out by Lu [59], indicating the need to establish solid experimental setups to validate the theoretical investigations in the current scientific literature. In this context, Berli [60], Boyko et al. [61] and Mei and Qian [62] realized theoretical investigations about the electrokinetic pumping of power law and viscoelastic fluids; here, they emphasized that the understanding and predictions of electroosmotic flows with non-Newtonian fluids are of practical importance for the experimental design as well as the operation of various micro/nanofluidic devices.

To the best authors’ knowledge, there are no studies in electroosmotic flows that simultaneously use the codeformational and corotational time derivatives in electroosmotic flows. Therefore, the objective of this research is to contribute to understanding the behavior of mixed electroosmotic/pressure driven flow of viscoelastic fluids between a parallel flat plates microchannel, based on the deformation and rotation that the material element experiences under flow. An analytical approximate solution for the velocity profile and flow rate using a regular perturbation scheme for small values of the viscoelastic parameter is obtained. The prediction of the flow field obtained in this work contributes to new findings that have not been reported previously, representing an important benchmark that will help in the design and control of experiments with viscoelastic fluids in microchannels.

## 2. Problem Formulation

### 2.1. Physical Model Description

**Figure 1:**
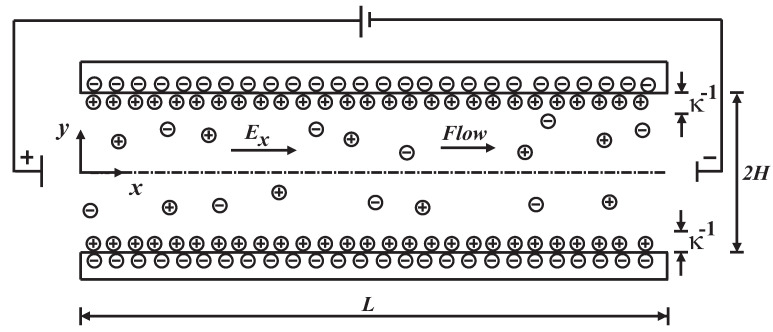
Schematic sketch of electroosmotic flow between two parallel flat plates.

The electroosmotic flow of an incompressible viscoelastic fluid in a microchannel formed by two parallel flat plates of height 2H and length *L* is considered. The origin of the rectangular coordinates system is placed at the *x*-symmetry axis of the microchannel and *y*-axis points out in the transverse direction, which is normal to the surface of the microchannel, as shown in Figure 1. The driving electroosmotic forces are provided by a constant electric field $E_{x}$ in the axial direction between the inlet and outlet of microchannel. A buffer solution of symmetrical electrolyte (z:z) is considered, at the channel walls constant zeta potentials are imposed. In the system, a high concentration of electric charges in the Debye length, $\kappa^{-1}$, inside of the electrical double layer (EDL) is presented.

To solve the problem, the following assumptions are considered:

- The flow is laminar and fully developed, V=(u(y),0,0).
- The fluid is incompressible.
- The fluid properties are constant. The temperature change of the fluid is less than 10 K [12] and throughout the flow domain, the temperature is uniform.
- The electrical double layers, do not overlap $H>\kappa^{-1}$.
- The electrolyte is symmetric, $z_{+}:z_{-}$.
- For the Poisson–Boltzmann solution, the electric potential in the vicinity of the wall is, ζ≤25 mV; therefore, the Debye–Hückel linearization can be used.

### 2.2. Governing and Constitutive Equations

The electroosmotic flow is governed by the continuity equation for an incompressible fluid,

(1) ∇·V=0,

and by the Cauchy momentum equation

(2) $$\rho \frac{DV}{Dt}=-\nabla \cdot \Pi +\rho_{e}E,$$

The $\frac{D}{Dt}$ represents the material derivative, ρ, V, ∇·, Π, $\rho_{e}$ and E are the fluid density, the velocity vector, the divergence operator, the total stress tensor, the electric charge density and the electric field vector, respectively. The total stress tensor is given by

(3) Π=pI+τ,

where *p*, I and τ are the pressure, the unit tensor and the extra stress tensor, respectively. According to the theory of electrostatics [2], the $\rho_{e}$ is governed by the Poisson equation

(4) $$\varepsilon \nabla^{2}\Phi =-\rho_{e},$$

where ε is the fluid dielectric permittivity and Φ is the total electric potential. To define the extra stress tensor, the viscoelastic Maxwell model used is

(5) $$\tau +\lambda \frac{D\tau }{Dt}=\eta_{0}D,$$

where λ, $\eta_{0}$ and $D=\nabla V+{\left(\nabla V\right)}^{T}$ are the fluid relaxation time, the zero-shear-rate viscosity and the rate of deformation tensor, respectively.

In this work, two time derivatives are used, the Oldroyd contravariant derivative, $\overset{\nabla }{\tau }$, also called codeformational derivative [38,39,40]

(6) $$\overset{\nabla }{\tau }=\frac{\partial }{\partial t}\tau +V\cdot \nabla \tau -\left[{\left(\nabla V\right)}^{T}\cdot \tau +\tau \cdot \left(\nabla V\right)\right],$$

and the Jaumann derivative, $\overset{\circ }{\tau }$, also called the corotational derivative [38,39,40] as follows

(7) $$\overset{\circ }{\tau }=\frac{\partial }{\partial t}\tau +V\cdot \nabla \tau -\frac{1}{2}\left[{\left(W\right)}^{T}\cdot \tau +\tau \cdot \left(W\right)\right],$$

where W is the vorticity tensor.

To establish the level of deformation/rotation that the fluid experiments with the flow, a configurational convective derivative, $\overset{⋄}{\tau }$, that contains both the Oldroyd and Jaumann time derivatives is proposed

(8) $$\overset{⋄}{\tau }=\frac{\partial }{\partial t}\tau +V\cdot \nabla \tau -\frac{1}{2}\left[{\left(W\right)}^{T}\cdot \tau +\tau \cdot \left(W\right)\right]+\left(1-\alpha \right)\left(\frac{1}{2}D\cdot \tau +\frac{1}{2}\tau \cdot D\right),$$

where α is the configurational parameter, which considers the level of deformation and rotation of the experimental fluid.

### 2.3. Poisson–Boltzmann Equation

The total electric potential for a long microchannel at any location in the system is given by a linear superposition of the applied external electric potential and the potential in the EDL, as $\Phi \left(x,y\right)\equiv \Phi =\psi \left(y\right)+\phi \left(x\right)$, where ψ(y) is the electric potential distribution within the EDL and $\phi \left(x\right)=\phi_{0}-xE_{x}$ is the external electric potential on the x− direction, $\phi_{0}$ is the electric potential at the inlet of the channel (x=0) and $E_{x}$ is the external electric field [2]. Due to the external electric field is independent of the position and constant along the axial direction the Poisson–Boltzmann equation is

(9) $$\frac{d^{2}\psi }{dy^{2}}=-\frac{\rho_{e}}{\varepsilon }.$$

Here, the electric charge density is given by $\rho_{e}=-2zen_{0}$sinh$\left(ze\psi /k_{B}T\right)$ where *z* is the valence of the ions, *e* is the electron charge, $n_{0}$ is the ionic density, $k_{B}$ is the Boltzmann constant and *T* is the absolute fluid temperature, respectively. Using the Debye–Hückel linearization, Equation (9) becomes

(10) $$\frac{d^{2}\psi }{dy^{2}}=\kappa^{2}\psi ,$$

where $\kappa^{2}=\left(2n_{0}z^{2}e^{2}/\varepsilon k_{B}T\right)$ is the Debye–Hückel parameter, related with the Debye length $\kappa^{-1}$ or EDL thickness [2]. The solution of Equation (10) requires the corresponding boundary conditions, zeta potential at the wall, $\psi_{\left(y=H\right)}=\zeta$ and at the symmetry plane/axis $\left(d\psi /dy\right){|}_{y=0}=0$, yielding

(11) $$\psi =\zeta \frac{\cosh\left(\kappa y\right)}{\cosh\left(\kappa H\right)},$$

and therefore $\rho_{e}$ for low surface potential is given by the combination of Equations (9)–(11), obtaining

(12) $$\rho_{e}=-\varepsilon \kappa^{2}\psi =-\varepsilon \kappa^{2}\zeta \frac{\cosh\left(\kappa y\right)}{\cosh\left(\kappa H\right)}.$$

### 2.4. Constitutive Equation

The symmetric extra stress tensor τ of the polymer solution is assumed. The extra stress tensor for the codeformational derivative can be obtained by inserting Equation (6) into Equation (5), and the normal $\tau_{xx}$, $\tau_{yy}$, $\tau_{zz}$ and shear $\tau_{xy}$ stresses can be expressed as

(13) $$\tau_{xx}=2\lambda \tau_{yx}\frac{\partial u}{\partial y},$$

(14) $$\tau_{yy}=0,$$

(15) $$\tau_{zz}=0$$

and

(16) $$\tau_{xy}=\eta_{0}\frac{\partial u}{\partial y},$$

where *u* is the fluid axial velocity. For the corotational derivative, the extra stress tensor can be obtained by inserting Equation (7) into Equation (5), which reduce to

(17) $$\tau_{xy}+\frac{1}{2}\lambda \left[\frac{\partial u}{\partial y}\left(\tau_{xx}-\tau_{yy}\right)\right]=\eta_{0}\frac{\partial u}{\partial y},$$

and the normal and shear stresses can be expressed as

(18) $$\tau_{xx}=\lambda \left(\frac{\partial u}{\partial y}\right)\tau_{xy},$$

(19) $$\tau_{xx}=-\tau_{yy},$$

(20) $$\tau_{zz}=0$$

and

(21) $$\tau_{xy}=\frac{\eta_{0}\frac{\partial u}{\partial y}}{\left[1+\lambda^{2}{\left(\frac{\partial u}{\partial y}\right)}^{2}\right]}.$$

On the other hand, the extra stress tensor for configurational convective derivative can be obtained by inserting Equation (8) into Equation (5), which reduce to

(22) $$\tau_{xy}+\frac{1}{2}\lambda \left\{\frac{\partial u}{\partial y}\left(\tau_{xx}-\tau_{yy}\right)-\left(1-\alpha \right)\left[\frac{\partial u}{\partial y}\left(\tau_{xx}+\tau_{yy}\right)\right]\right\}=\eta_{0}\frac{\partial u}{\partial y},$$

and the normal and shear stresses can be expressed as

(23) $$\tau_{xx}=\lambda \frac{\partial u}{\partial y}\tau_{yx}\left(2-\alpha \right),$$

(24) $$\tau_{yy}=-\lambda \frac{\partial u}{\partial y}\tau_{xy}\left(\alpha \right),$$

(25) $$\tau_{zz}=0$$

and

(26) $$\tau_{xy}=\frac{\eta_{0}\frac{\partial u}{\partial y}}{\left[1+\lambda^{2}{\left(\frac{\partial u}{\partial y}\right)}^{2}\left(2\alpha -\alpha^{2}\right)\right]}.$$

The principal advantage of the global model represented by Equation (22) is that it is possible to work with viscoelastic fluids using the codeformational (α=0), corotational (α=1) or the mixture of both (0≤α≤1) time derivatives. The normal and shear stresses, the first normal stress difference $N_{1}$ and the first normal stress coefficient $\Psi_{1}$ of the Maxwell model using the different time derivatives are summarized in Table 1, where $\dot{\gamma }=\frac{\partial u}{\partial y}$ is the shear rate. It is important to mention that an adequate constitutive equation for viscoelastic fluids can predict the rheological properties presented in Table 1. These properties can be determined experimentally by rheometric measurements to adjust the model for a specific fluid.

### 2.5. Cauchy Momentum Equation

According to the assumptions presented in Section 2.1, the conservation momentum equation is given by

(27) $$\frac{\partial }{\partial y}\tau_{xy}=\frac{dp}{dx}-\rho_{e}E_{x},$$

where $dp/dx\equiv p{,}_{x}$. Inserting Equation (12) into Equation (27) and upon integration respect to *y*,

(28) $$\tau_{xy}=p{,}_{x}y+\varepsilon \kappa \zeta E_{x}\frac{\sinh\left(\kappa y\right)}{\cosh\left(\kappa H\right)}+c,$$

where *c* is an integration constant. Introducing Equation (26) into Equation (28), a nonlinear differential equation is obtained

(29) $$\frac{\frac{\partial u}{\partial y}}{\left[1+\lambda^{2}{\left(\frac{\partial u}{\partial y}\right)}^{2}\left(2\alpha -\alpha^{2}\right)\right]}=\frac{1}{\eta_{0}}\left(p{,}_{x}y+\varepsilon \kappa \zeta E_{x}\frac{\sinh\left(\kappa y\right)}{\cosh\left(\kappa H\right)}+c\right).$$

The corresponding boundary conditions for Equation (29) are the no-slip boundary condition at the channel walls and the symmetry boundary condition at the centerline of the microchannel, respectively, as

(30) $$u\left(y=H\right)=0$$

and

(31) $${\left.\frac{\partial u}{\partial y}\right|}_{\left(y=0\right)}=0.$$

### 2.6. Dimensionless Equations

To obtain the dimensionless set of Equations (29)–(31), the following scale factors are used: $\bar{u}=u/u_{sh},\bar{y}=y/H$, ${\bar{\tau }}_{xx}=\tau_{xx}H/\eta_{0}u_{sh}$, ${\bar{\tau }}_{yy}=\tau_{yy}H/\eta_{0}u_{sh}$ and ${\bar{\tau }}_{xy}=\tau_{xy}H/\eta_{0}u_{sh}$. First, the following expression is yielded:

(32) $$\frac{\frac{\partial \bar{u}}{\partial \bar{y}}}{\left[1+De_{sh}^{2}{\left(\frac{\partial \bar{u}}{\partial \bar{y}}\right)}^{2}\left(2\alpha -\alpha^{2}\right)\right]}=\Gamma \bar{y}-\bar{\kappa }\frac{\sinh\left(\bar{\kappa }\bar{y}\right)}{\cosh\left(\bar{\kappa }\right)}+C,$$

where $u_{sh}=-\varepsilon \zeta E_{x}/\eta_{0}$ is the characteristic Helmholtz–Smoluchowski electroosmotic velocity; $De_{sh}=\lambda u_{sh}/H$ is the Deborah number based on $u_{sh}$ and $\Gamma =-\left(H^{2}/\varepsilon \zeta \right)\left(p{,}_{x}/E_{x}\right)$, which represents the ratio of pressure to electroosmotic driving forces; and $\bar{\kappa }=H/\kappa^{-1}$ is the ratio between the microchannel half-height to the Debye length or electrokinetic parameter.

Secondly, the dimensionless boundary conditions are obtained, respectively, as

(33) $$\bar{u}\left(\bar{y}=1\right)=0$$

and

(34) $${\left.\frac{\partial \bar{u}}{\partial \bar{y}}\right|}_{\left(\bar{y}=0\right)}=0.$$

In Equation (32), $De_{sh}^{2}$ is replaced by the parameter ϵ, $A=-\frac{\bar{\kappa }\sinh\left(\bar{\kappa }\bar{y}\right)}{\cosh\left(\bar{\kappa }\right)}$ and *C* is a dimensionless integration constant. Therefore, Equation (32) can be rewritten as

(35) $$\frac{\partial \bar{u}}{\partial \bar{y}}=\left[1+\epsilon {\left(\frac{\partial \bar{u}}{\partial \bar{y}}\right)}^{2}\left(2\alpha -\alpha^{2}\right)\right]\left(\Gamma \bar{y}+A+C\right).$$

### 2.7. Approximate Solution

To determine the solution of the electroosmotic flow, Equations (33)–(35) are solved by asymptotic technique. Firstly, the perturbation parameter is identified, in this case the Deborah number is replace by ϵ. The solution is restricted to small values of ϵ (low viscoelastic fluids); in the limit ϵ→0, the Newtonian solution is obtained. Equation (35) is solved applied a regular perturbation method for small values of ϵ. For this purpose, the following expansions are suggested [40]

(36) $$\bar{u}={\bar{u}}_{0}+\epsilon {\bar{u}}_{1}+...,$$

(37) $$A=A_{0}+\epsilon A_{1}+...$$

and

(38) $$C=C_{0}+\epsilon C_{1}+...,$$

with the use of Equations (36)–(38), Equation (35) describes the mixed electroosmotic/pressure driven flow presented in the last section take the form

(39) $$\begin{array}{c} \frac{\partial \bar{u}}{\partial \bar{y}}+\epsilon \frac{\partial {\bar{u}}_{1}}{\partial \bar{y}}+...=\left[1+\epsilon {\left(\frac{\partial {\bar{u}}_{0}}{\partial \bar{y}}+\epsilon \frac{\partial {\bar{u}}_{1}}{\partial \bar{y}}+...\right)}^{2}\left(2\alpha -\alpha^{2}\right)\right]\\ \left[\bar{y}\Gamma +A_{0}+\epsilon A_{1}+...+C_{0}+\epsilon C_{1}+...\right] \end{array}$$

and the boundary conditions in Equations (33) and (34)

(40) $${\bar{u}}_{0}+\epsilon {\bar{u}}_{1}+...=0$$

and

(41) $$\frac{\partial {\bar{u}}_{0}}{\partial \bar{y}}+\epsilon \frac{\partial {\bar{u}}_{1}}{\partial \bar{y}}+...=0$$

for the terms of zeroth power $\left(\epsilon^{0}\right)$, Equation (39) reduces to

(42) $$\frac{\partial {\bar{u}}_{0}}{\partial \bar{y}}=\bar{y}\Gamma +A_{0}+C_{0},$$

and the corresponding boundary conditions from Equations (40) and (41) yield

(43) $${\bar{u}}_{0}=0$$

and

(44) $$\frac{\partial {\bar{u}}_{0}}{\partial \bar{y}}=0.$$

Integrating in the axial direction the Equation (42) and applying the boundary conditions in Equations (43) and (44), the dimensionless velocity profile for the mixed electroosmotic/pressure driven flows of Newtonian fluids is obtained as

(45) $${\bar{u}}_{0}=\frac{1}{2}\Gamma \left({\bar{y}}^{2}-1\right)+1-\frac{\cosh\left(\bar{\kappa }\bar{y}\right)}{\cosh\left(\bar{\kappa }\right)}$$

For terms of first power $\left(\epsilon \right)$, Equation (39) reduces to

(46) $$\frac{\partial {\bar{u}}_{1}}{\partial \bar{y}}=A_{1}+C_{1}+{\left(\frac{\partial {\bar{u}}_{0}}{\partial \bar{y}}\right)}^{2}\left(2\alpha -\alpha^{2}\right)\left(\bar{y}\Gamma +A_{0}+C_{0}\right),$$

and the corresponding boundary conditions from Equations (40) and (41) yield

(47) $${\bar{u}}_{1}=0$$

and

(48) $$\frac{\partial {\bar{u}}_{1}}{\partial \bar{y}}=0,$$

from Equations (46)–(48), the solution for the velocity component $u_{1}$ is obtained

(49) $$\begin{array}{ccc} {\bar{u}}_{1} & = & -\left(\frac{\cosh\left(\bar{\kappa }\bar{y}\right)}{\cosh\left(\bar{\kappa }\right)}-1\right)\\ & + & \frac{1}{4}\left(2\alpha -\alpha^{2}\right)\Gamma^{3}\left({\bar{y}}^{4}-1\right)\\ & - & 3\left(2\alpha -\alpha^{2}\right)\Gamma^{2}\left({\bar{y}}^{2}\frac{\cosh\left(\bar{\kappa }\bar{y}\right)}{\cosh\left(\bar{\kappa }\right)}-1\right)\\ & + & 6\left(2\alpha -\alpha^{2}\right)\frac{\Gamma^{2}}{\bar{\kappa }}\left(\bar{y}\frac{\sinh\left(\bar{\kappa }\bar{y}\right)}{\cosh\left(\bar{\kappa }\right)}-\frac{\sinh\left(\bar{\kappa }\right)}{\cosh\left(\bar{\kappa }\right)}\right)\\ & - & 6\left(2\alpha -\alpha^{2}\right)\frac{\Gamma^{2}}{{\bar{\kappa }}^{2}}\left(\frac{\cosh\left(\bar{\kappa }\bar{y}\right)}{\cosh\left(\bar{\kappa }\right)}-1\right)\\ & + & \frac{3}{4}\left(2\alpha -\alpha^{2}\right)\Gamma \bar{\kappa }\left(\bar{y}\frac{\sinh(2\bar{\kappa }\bar{y})}{\cosh^{2}\left(\bar{\kappa }\right)}-\frac{\sinh(2\bar{\kappa })}{\cosh^{2}\left(\bar{\kappa }\right)}\right)\\ & - & \frac{3}{8}\left(2\alpha -\alpha^{2}\right)\Gamma \left(\frac{\cosh\left(2\bar{\kappa }\bar{y}\right)}{\cosh^{2}\left(\bar{\kappa }\right)}-\frac{\cosh\left(2\bar{\kappa }\right)}{\cosh^{2}\left(\bar{\kappa }\right)}\right)\\ & - & \frac{3}{4}\left(2\alpha -\alpha^{2}\right)\Gamma {\bar{\kappa }}^{2}\left(\frac{{\bar{y}}^{2}}{\cosh^{2}\left(\bar{\kappa }\right)}-\frac{1}{\cosh^{2}\left(\bar{\kappa }\right)}\right)\\ & - & \frac{1}{3}\left(2\alpha -\alpha^{2}\right){\bar{\kappa }}^{2}\left(\frac{\cosh^{3}\left(\bar{\kappa }\bar{y}\right)}{\cosh^{3}\left(\bar{\kappa }\right)}-1\right)\\ & + & \left(2\alpha -\alpha^{2}\right){\bar{\kappa }}^{2}\left(\frac{\cosh\left(\bar{\kappa }\bar{y}\right)}{\cosh^{3}\left(\bar{\kappa }\right)}-\frac{1}{\cosh^{2}\left(\bar{\kappa }\right)}\right). \end{array}$$

The resulting velocity profile from Equation (36), and by considering the zeroth and first order for the perturbation parameter ϵ in the approximate solution, is the following

(50) $$\begin{array}{ccc} \bar{u} & = & \frac{1}{2}\Gamma \left({\bar{y}}^{2}-1\right)+1-\frac{\cosh\left(\bar{\kappa }\bar{y}\right)}{\cosh\left(\bar{\kappa }\right)}\\ & - & \epsilon \left(\frac{\cosh\left(\bar{\kappa }\bar{y}\right)}{\cosh\left(\bar{\kappa }\right)}-1\right)\\ & + & \frac{\epsilon }{4}\left(2\alpha -\alpha^{2}\right)\Gamma^{3}\left({\bar{y}}^{4}-1\right)\\ & - & 3\epsilon \left(2\alpha -\alpha^{2}\right)\Gamma^{2}\left({\bar{y}}^{2}\frac{\cosh\left(\bar{\kappa }\bar{y}\right)}{\cosh\left(\bar{\kappa }\right)}-1\right)\\ & + & 6\epsilon \left(2\alpha -\alpha^{2}\right)\frac{\Gamma^{2}}{\bar{\kappa }}\left(\bar{y}\frac{\sinh\left(\bar{\kappa }\bar{y}\right)}{\cosh\left(\bar{\kappa }\right)}-\frac{\sinh\left(\bar{\kappa }\right)}{\cosh\left(\bar{\kappa }\right)}\right)\\ & - & 6\epsilon \left(2\alpha -\alpha^{2}\right)\frac{\Gamma^{2}}{{\bar{\kappa }}^{2}}\left(\frac{\cosh\left(\bar{\kappa }\bar{y}\right)}{\cosh\left(\bar{\kappa }\right)}-1\right)\\ & + & \frac{3\epsilon }{4}\left(2\alpha -\alpha^{2}\right)\Gamma \bar{\kappa }\left(\bar{y}\frac{\sinh(2\bar{\kappa }\bar{y})}{\cosh^{2}\left(\bar{\kappa }\right)}-\frac{\sinh(2\bar{\kappa })}{\cosh^{2}\left(\bar{\kappa }\right)}\right)\\ & - & \frac{3\epsilon }{8}\left(2\alpha -\alpha^{2}\right)\Gamma \left(\frac{\cosh\left(2\bar{\kappa }\bar{y}\right)}{\cosh^{2}\left(\bar{\kappa }\right)}-\frac{\cosh\left(2\bar{\kappa }\right)}{\cosh^{2}\left(\bar{\kappa }\right)}\right)\\ & - & \frac{3\epsilon }{4}\left(2\alpha -\alpha^{2}\right)\Gamma {\bar{\kappa }}^{2}\left(\frac{{\bar{y}}^{2}}{\cosh^{2}\left(\bar{\kappa }\right)}-\frac{1}{\cosh^{2}\left(\bar{\kappa }\right)}\right)\\ & - & \frac{\epsilon }{3}\left(2\alpha -\alpha^{2}\right){\bar{\kappa }}^{2}\left(\frac{\cosh^{3}\left(\bar{\kappa }\bar{y}\right)}{\cosh^{3}\left(\bar{\kappa }\right)}-1\right)\\ & + & \epsilon \left(2\alpha -\alpha^{2}\right){\bar{\kappa }}^{2}\left(\frac{\cosh\left(\bar{\kappa }\bar{y}\right)}{\cosh^{3}\left(\bar{\kappa }\right)}-\frac{1}{\cosh^{2}\left(\bar{\kappa }\right)}\right). \end{array}$$

The dimensionless flow rate $\bar{Q}$ can be determined from integration of the velocity profile of Equation (50). The velocity profile can be simplified due to the electric double layer being much smaller than the microchannel half-height ($10\leq \bar{\kappa }\leq 100$), $\cosh\left(\bar{\kappa }\right)\gg 1$, and therefore $\tanh\left(\bar{\kappa }\right)\approx 1$; the dimensionless flow rate becomes

(51) $$\begin{array}{ccc} \bar{Q} & = & 2\int_{0}^{1}\bar{u}d\bar{y}=2-\frac{2}{3}\Gamma -\frac{2}{\bar{\kappa }}\\ & - & 2\epsilon \left(\frac{1}{\bar{\kappa }}-1\right)\\ & - & \frac{2}{5}\epsilon \left(2\alpha -\alpha^{2}\right)\Gamma^{3}-6\epsilon \left(2\alpha -\alpha^{2}\right)\Gamma^{2}\left(\frac{1}{\bar{\kappa }}-\frac{2}{{\bar{\kappa }}^{2}}+\frac{2}{{\bar{\kappa }}^{3}}-1\right)\\ & + & 12\epsilon \left(2\alpha -\alpha^{2}\right)\Gamma^{2}\left(\frac{1}{{\bar{\kappa }}^{2}}-\frac{1}{{\bar{\kappa }}^{3}}-\frac{1}{\bar{\kappa }}\right)-12\epsilon \left(2\alpha -\alpha^{2}\right)\Gamma^{2}\left(\frac{1}{{\bar{\kappa }}^{3}}-\frac{1}{{\bar{\kappa }}^{2}}\right)\\ & + & \frac{3}{2}\epsilon \left(2\alpha -\alpha^{2}\right)\Gamma \left(\frac{1}{2}\frac{\cosh\left(2\bar{\kappa }\right)}{\cosh^{2}\left(\bar{\kappa }\right)}-\frac{1}{4\bar{\kappa }}\frac{\sinh\left(2\bar{\kappa }\right)}{\cosh^{2}\left(\bar{\kappa }\right)}-\bar{\kappa }\frac{\sinh\left(2\bar{\kappa }\right)}{\cosh^{2}\left(\bar{\kappa }\right)}\right)\\ & - & \frac{3}{4}\epsilon \left(2\alpha -\alpha^{2}\right)\Gamma \left(\frac{1}{2\bar{\kappa }}\frac{\sinh\left(2\bar{\kappa }\right)}{\cosh^{2}\left(\bar{\kappa }\right)}-\frac{\cosh\left(2\bar{\kappa }\right)}{\cosh^{2}\left(\bar{\kappa }\right)}\right)\\ & + & \epsilon \left(2\alpha -\alpha^{2}\right)\Gamma \frac{{\bar{\kappa }}^{2}}{\cosh^{2}\left(\bar{\kappa }\right)}-\frac{2}{3}\epsilon \left(2\alpha -\alpha^{2}\right)\left(\frac{\bar{\kappa }}{\cosh^{2}\left(\bar{\kappa }\right)}-{\bar{\kappa }}^{2}\right)\\ & - & \frac{2}{9}\epsilon \left(2\alpha -\alpha^{2}\right)\bar{\kappa }+2\epsilon \left(2\alpha -\alpha^{2}\right)\left(\frac{\bar{\kappa }}{\cosh^{2}\left(\bar{\kappa }\right)}-\frac{{\bar{\kappa }}^{2}}{\cosh^{2}\left(\bar{\kappa }\right)}\right). \end{array}$$

## 3. Results and Discussion

Different time derivatives into the Maxwell model for an electroosmotic flow in a parallel plates microchannel are analyzed. The dimensionless parameters used to validate the approximate solution in Equation (50) were obtained by a suitable combination of the following parameters: 0.1≤H≤10μm, $1\leq \kappa^{-1}\leq 300$ nm, $E_{x}∼$$10^{4}$ Vm ${}^{-1}$, ζ≤25 mV, 7×$10^{-10}\leq \varepsilon \leq 10^{-9}$ CV${}^{-1}$m${}^{-1}$, $10^{-4}\leq \eta_{0}\leq 10^{-2}$ kgm${}^{-1}$s${}^{-1}$.

**Figure 2:**
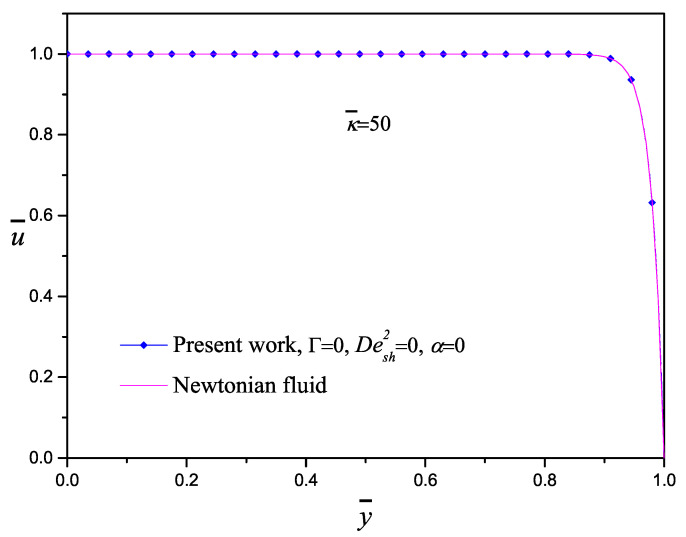
Comparison of the analytical solution and the approximate solution (present work) for an electroosmotic flow.

Equation (45) describes the axial velocity of an electrolyte solution by two contributions, the first one due to an imposed pressure gradient with a characteristic parabolic velocity profile (called Poiseuille flow) and the second one due to an imposed electrical field usually called electroosmotic flow with a characteristic fairly flat velocity profile (called plug flow). Figure 2 shows the comparison of the velocity profiles of an electroosmotic flow obtained in this work and those carried out by Masliyah and Bhattacharjee [2] using the same value of the electrokinetic parameter $\bar{\kappa }=50$. To compare the results, the parameters $\Gamma =0,De_{sh}^{2}=0,\alpha =0$ were selected, where the pressure gradient and the viscoelasticity effects are neglected. Therefore, the classical plug-like profile for both models is exhibited; here, an excellent agreement between the approximate solution and the exact solution is obtained.

**Figure 3:**
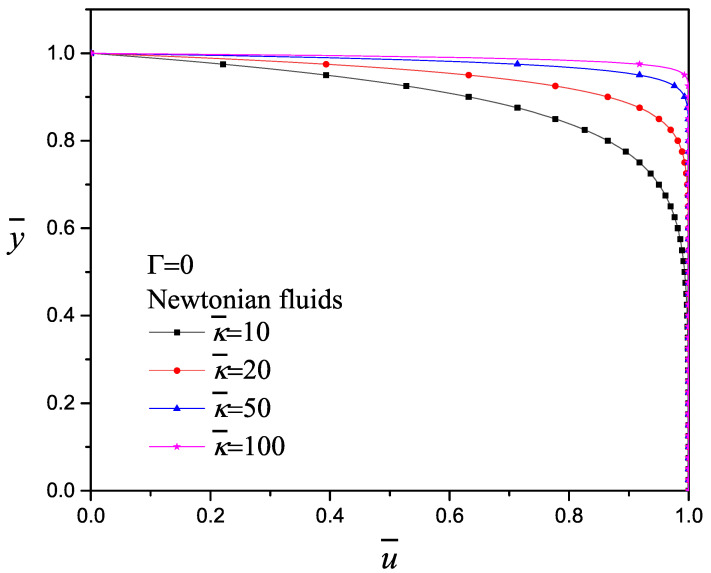
Effect of the electrokinetic parameter on the velocity profile with Γ=0 and $De_{sh}^{2}=0$.

The electrokinetic parameter $\bar{\kappa }$ is defined as the ratio of the microchannel half-height to the Debye length and considering the assumption that $H>\kappa^{-1}$. It means that the size of the EDL or region of excess charge is relatively small. The effect of the electrokinetic parameter $\bar{\kappa }$ on the velocity profile for a purely electroosmotic flow with Γ=0 and $De_{sh}^{2}=0$ is shown in Figure 3: lower $\bar{\kappa }$ for Poiseuille-like and higher $\bar{\kappa }$ for plug-like flow. The same behavior was reported by Afonso et al. [23]. Dutta et al. [63] reported a typical example for a 0.1 mM buffer solution in a channel with $\bar{\kappa }=100$ and ζ=25 mV and determined the Debye length in the order of 30 nm for a channel height 6 μm; for 1 mM concentration the Debye length becomes 10 nm for a channel height of 2 μm. According to Debye–Hückel approximation, the minimum of $\bar{\kappa }≅$ 10, while lower values are not compatible. 

**Figure 4:**
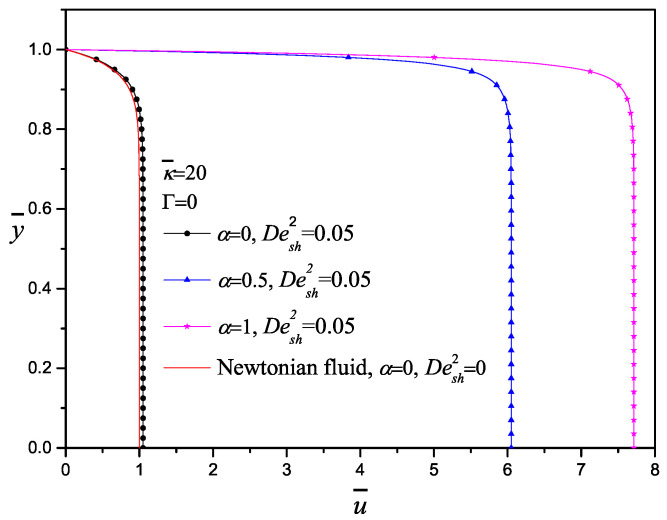
Effect of the codeformational (α=0), mixture (α=0.5) and corotational (α=1) derivatives on the velocity profile with $\bar{\kappa }=20,\Gamma =0,De_{sh}^{2}=0.05$.

In Figure 4, the dimensionless velocity profiles for the purely electroosmotic flow (Γ=0) varying the configurational parameter α are shown; no significant changes between the Newtonian model with α=0 and $De_{sh}^{2}=0$ and the codeformational model with α=0 and $De_{sh}^{2}=0.05$ are appreciated. On the other hand, by increasing α, the corotational model is taking into account increasing almost eight times the velocity magnitude for α=1. It is important to mention that the corotational derivative considers only rotation of the material element with the flow field, which is possible close to the wall where the velocity gradient exists. This situation is possible for shear thinning fluids, which experience less resistant to flow. This behavior with viscoelastic fluids is presented when the polymer chains organize to flow and the energy required is less.

**Figure 5:**
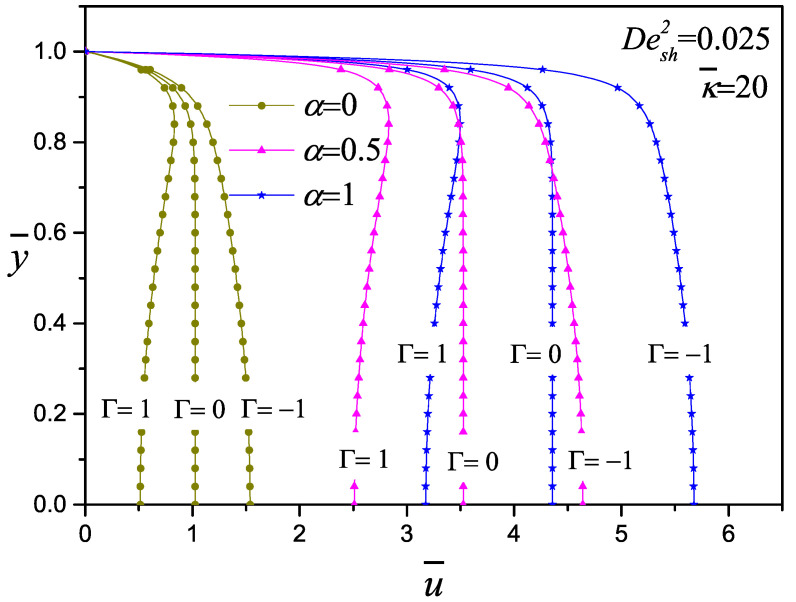
Effect of the pressure gradient (Γ=1,0,−1) on the velocity profile with $\bar{\kappa }=20$ and $De_{sh}^{2}=0.025$ for the codeformational (α=0), mixture (α=0.5) and corotational (α=1) models.

In Figure 5, the influence of the pressure gradient on the dimensionless velocity of viscoelastic fluids with $De_{sh}^{2}=0.025$ and $\bar{\kappa }=20$ is shown; Γ represents the ratio of pressure to electroosmotic driving forces and the electrokinetic parameter that for this cases are equally important. Depending on the direction of the gradient pressure, concave and convex Poiseuille velocity profiles are obtained; the shapes of these profiles were reported by Afonso et al. [23] when α=0 for a Newtonian fluid. The three cases have the same behavior: from codeformational derivative to corotational derivative, an important increment of the velocity magnitude is obtained. Most of the viscoelastic fluids deform and rotate under flow and the value of the configurational parameter has to be determined experimentally for specific viscoelastic fluid. For mixed electroosmotic and pressure driving flow, the resulting velocity profile is the superimposed effect of both electroosmotic and pressure gradient flow phenomena. It is important to note that negative and positive velocities are presented in the channel, depending on Γ>0 or Γ<0, respectively. When Γ is opposite to the flow, the highest velocity is close to the wall.

**Figure 6:**
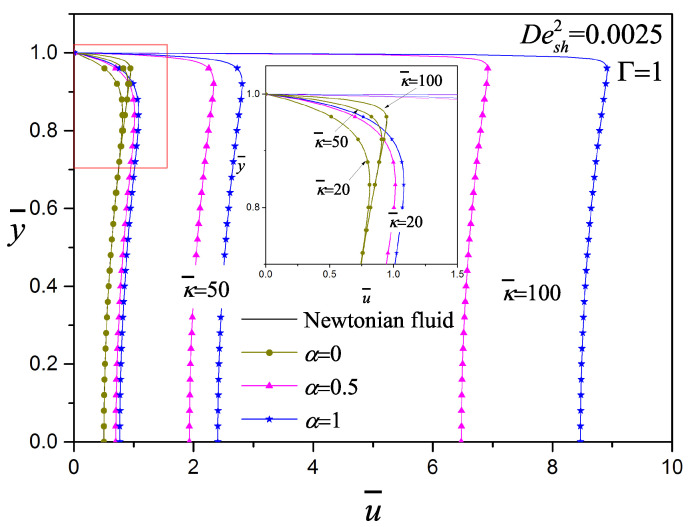
Effect of the electrokinetic parameter ($\bar{\kappa }=20,50,100$) with $De_{sh}^{2}=0.0025$ and Γ=1 for the codeformational (α=0), mixture (α=0.5) and corotational (α=1) models.

In Figure 6, the effect of the electrokinetic parameter $\bar{\kappa }$ on the dimensionless velocity of viscoelastic fluids with $De_{sh}^{2}=0.0025$ and Γ=1 is presented; by increasing $\bar{\kappa }$ the velocity profile changes from Poiseuille to Plug flow, which is the same behavior analyzed and presented in Figure 2 and Figure 3. This behavior is better appreciated when α=0 (the inset figure), which is very similar to the Newtonian case, where close to the wall the velocity becomes flatter for larger $\bar{\kappa }$. The velocity magnitude increases considerably with the following combination of parameters; the viscoelasticity of the fluid, high electrokinetic parameter ($\bar{\kappa }=100$) and α≠0. Afonso et al. [23] obtained analytical solutions for the flow of viscoelastic fluids using the PTT model, They mentioned that the $\bar{\kappa }$ effects on the velocity profile is restricted to a narrow region, the effective EDL thickness: higher values of $\bar{\kappa }$ lead to thinner EDL and consequently higher velocity gradients.

**Figure 7:**
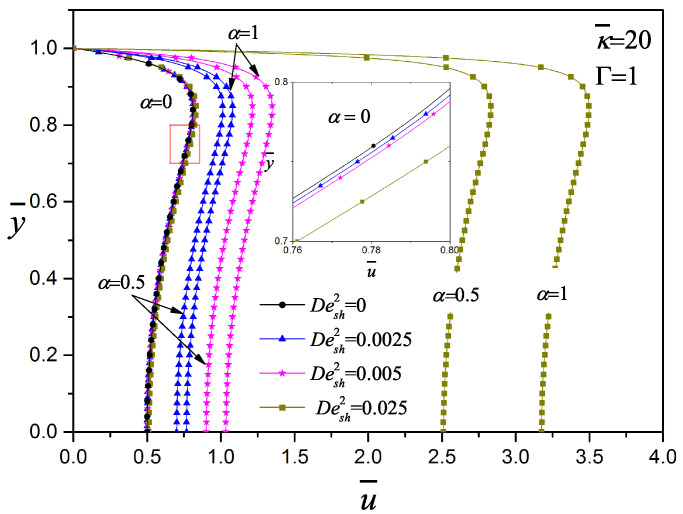
Influence of the viscoelasticity on the velocity profile with $\bar{\kappa }=20$ and Γ=1, for the codeformational (α=0), mixture (α=0.5) and corotational (α=1) models.

The internal micro-structure of viscoelastic fluids interact in a complex process with electric fields and surfaces, leading either to adsorption or wall-depletion, as described in [51,57]. In Figure 7, the effect of the viscoelasticity on the dimensionless velocity with $\bar{\kappa }=20$ and Γ=1 is exhibited. When the $De_{sh}^{2}$ increases, the velocity plateau also increases significantly; the same results were reported by Afonso et al. [23] using the simplified PTT model. Ferras et al. [51] analyzed the effects of the solvent to polymer viscosity ratio and viscoelasticity using the simplified Phan–Thien–Tanner model with linear stress coefficient function; in their work, the Debye–Hückel approximation and the electrokinetic parameter of ($\bar{\kappa }=20$) were used. They reported that increasing the viscoelasticity leads to an increase of the dimensionless velocity, which is more intense for intermediate values of viscosity ratio (Newtonian viscosity/(Newtonian viscosity + polymer viscosity)). They also concluded that the viscoelastic parameter quantifies not only the elasticity via the normal stresses, but measures the shear-thinning of the viscosity that directly affects the velocity profile as well. An increase of the maximum velocity can be observed by increasing the ratio of the viscosities as well as the fluid shear-thinning. In this context, the configurational parameter α of the present work performs the same function as the viscosity ratio together with the fluid viscoelasticity.

**Figure 8:**
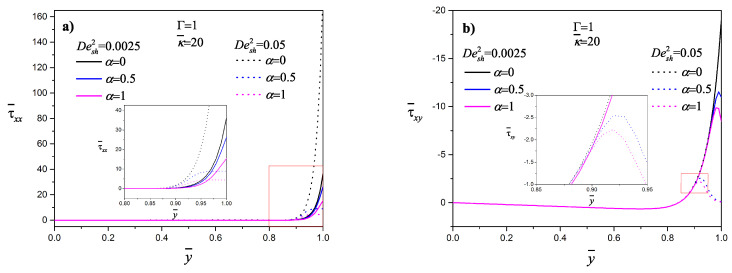
Dimensionless (**a**) normal and (**b**) shear stresses as a function of the position for the codeformational (α=0), mixture (α=0.5) and corotational (α=1) models, for $De_{sh}^{2}=0.0025$ and 0.05.

The normal and shear stress profiles as a function of the transversal position for two values of $De_{sh}^{2}$ are shown in Figure 8a,b, respectively. In the channel central region, the shear and normal stresses are practically zero; the presence of both stresses is only close to the wall, being higher for lower $De_{sh}^{2}$ for α≠0. Ferras et al. [51] found the same behavior using the simplified Phan–Thien–Tanner model with a linear stress coefficient function for the polymer contribution plus a Newtonian solvent. For the codeformational time derivative (α=0), both the shear and normal stresses are higher with increasing viscoelasticity. For the codeformational time derivative (α=0), both the shear and normal stresses are higher with increasing viscoelasticity. Afonso et al. [23] reported that the normal stresses increase linearly with the Deborah number regardless of $\bar{\kappa }$.

**Figure 9:**
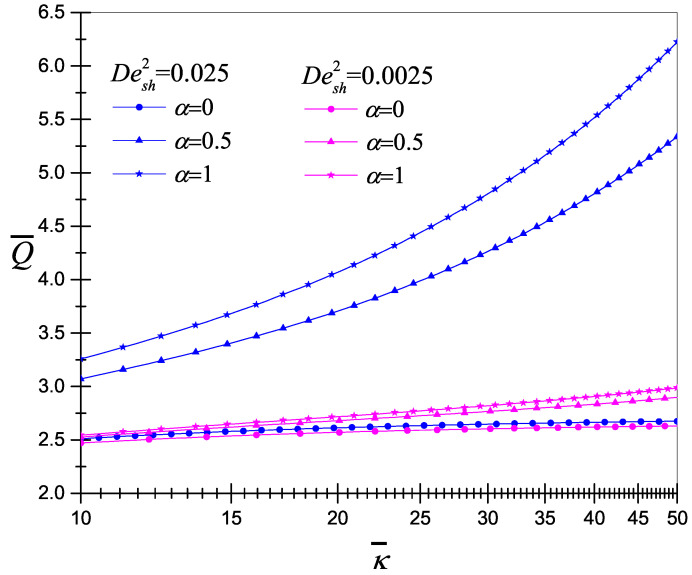
The volumetric flow rate as a function of the electrokinetic parameter for the codeformational (α=0), mixture (α=0.5) and corotational (α=1) models, for $De_{sh}^{2}=0.0025$ and 0.025.

Finally, the dimensionless volumetric flow rate as a function of the electrokinetic parameter, for two viscoelastic fluids with $De_{sh}^{2}=0.0025$ and $De_{sh}^{2}=0.025$, is presented in Figure 9. The flow rate increases almost linearly with $\bar{\kappa }$; the viscoelastic effect increases considerably the flow rate. The flow rate depends on both $\bar{\kappa }$ and the viscoelasticity; with these two parameters, the flow rate can be controlled. Characteristics of electroosmotic flows of non-Newtonian fluids of viscoelastic have been investigated theoretically [17,23,25,37,49,51]; these investigations unanimously revealed a common feature that the flow pattern as well as volumetric flow rate of electroosmosis is significantly affected due to the existence of fluid viscoelasticity [17].

## 4. Conclusions

In this work, an analytical approximate solution of mixed electroosmotic pressure driven flows of viscoelastic fluids between parallel plates was obtained. The Oldroyd and Jaumann time derivatives in the Maxwell model were used. The most important findings of this research work are:

- The model was compared and validated with results published in the specialized literature.
- The approximate solution is only valid for low viscoelastic fluids.
- The approximate solution adequately captures the physics of the problem; it can be adjusted with experimental data of a specific viscoelastic fluid.
- The electrokinetic parameter changes the velocity profile, lower $\bar{\kappa }$ for Poiseuille-like and higher $\bar{\kappa }$ for plug-like flow, as is reported by other models.
- Higher values of $\bar{\kappa }$ lead to thinner EDL and consequently higher velocity gradients.
- The direction of gradient pressure modifies the velocity profile to concave or convex, as reported in the literature.
- An increment of the viscoelasticity fluid generates an increase of the velocity and the flow rate.
- The presence of the shear and normal stresses is only close to the wall.
- The configurational parameter α performs the same function as the viscosity ratio used in other models; this parameter directly affects the velocity field magnitude.
- For practical applications, the volumetric flow rate can be controlled with two parameters, namely the gradient pressure and the electrokinetic parameter, once the fluid has been rheologically characterized.

This model can be used as a tool to understand the flow behavior of low viscoelastic fluids, which translate, deform and rotate under flow. There are theoretical investigations and models in microfluidics that require establishing solid experimental setups to validate and improve them.

## Figures and Tables

**Table 1:** Principal variables of the Maxwell model using different time derivatives.

Variables	Oldroyd	Jaumann	Configurational (Mixed)
$\tau_{xx}$	$2\lambda \eta_{0}{\dot{\gamma }}^{2}$	$\frac{\lambda \eta_{0}{\dot{\gamma }}^{2}}{1+\lambda^{2}{\dot{\gamma }}^{2}}$	$\frac{\lambda \eta_{0}{\dot{\gamma }}^{2}\left(2-\alpha \right)}{1+\lambda^{2}{\dot{\gamma }}^{2}\left(2\alpha -\alpha^{2}\right)}$
$\tau_{yy}$	0	$-\frac{\lambda \eta_{0}{\dot{\gamma }}^{2}}{1+\lambda^{2}{\dot{\gamma }}^{2}}$	$-\frac{\lambda \eta_{0}{\dot{\gamma }}^{2}\alpha }{1+\lambda^{2}{\dot{\gamma }}^{2}\left(2\alpha -\alpha^{2}\right)}$
$\tau_{xy}$	$\eta_{0}\dot{\gamma }$	$\frac{\eta_{0}\dot{\gamma }}{1+\lambda^{2}{\dot{\gamma }}^{2}}$	$\frac{\eta_{0}\dot{\gamma }}{1+\lambda^{2}{\dot{\gamma }}^{2}\left(2\alpha -\alpha^{2}\right)}$
$N_{1}=\tau_{xx}-\tau_{yy}$	$2\lambda \eta_{0}{\dot{\gamma }}^{2}$	$\frac{2\lambda \eta_{0}{\dot{\gamma }}^{2}}{1+\lambda^{2}{\dot{\gamma }}^{2}}$	$\frac{2\lambda \eta_{0}{\dot{\gamma }}^{2}}{1+\lambda^{2}{\dot{\gamma }}^{2}\left(2\alpha -\alpha^{2}\right)}$
$\Psi_{1}=\frac{N_{1}}{{\dot{\gamma }}^{2}}$	$2\lambda \eta_{0}$	$\frac{2\lambda \eta_{0}}{1+\lambda^{2}{\dot{\gamma }}^{2}}$	$\frac{2\lambda \eta_{0}}{1+\lambda^{2}{\dot{\gamma }}^{2}\left(2\alpha -\alpha^{2}\right)}$

## Nomenclature

$De_{sh}$	Deborah number
*e*	electron charge, 1.602 × 10${}^{-19}$ C
$E_{x}\;$	external electric field, Vm${}^{-1}$
*H*	microchannel half-height, m
$k_{B}$	Boltzmann constant, 1.381 × 10${}^{-23}$ JK${}^{-1}$
*L*	microchannel length, m
$n_{0}$	ionic density, m${}^{-3}$
$N_{1}$	first normal stress difference, kgm${}^{-1}$s${}^{-2}$
*p*	pressure, kgm${}^{-1}$s${}^{-2}$
*t*	time, s
*T*	absolute temperature, K
*u*	axial velocity, ms${}^{-1}$
$u_{sh}$	Helmholtz-Smoluchowski electroosmotic velocity, ms${}^{-1}$
*x*	axial coordinate, m
*y*	transverse coordinate, m
*z*	valence of the ions
$\bar{Q}$	dimensionless flow rate
$\bar{u}$	dimensionless fluid axial velocity
$\bar{y}$	dimensionless transverse coordinate

Tensorsandvectors
D	rate of deformation tensor, s${}^{-1}$
E	electric field vector, Vm${}^{-1}$
I	unit tensor
V	velocity vector, ms${}^{-1}$
W	vorticity tensor
τ	extra stress tensor, kgm${}^{-1}$s${}^{-2}$
Π	total stress tensor, kgm${}^{-1}$s${}^{-2}$
Φ	total electric potential, V

Greeksymbols
α	configurational parameter
χ	dimensionless axial coordinate
$\eta_{0}$	zero-shear-rate viscosity, kgm${}^{-1}$s${}^{-1}$
Γ	ratio of pressure to electroosmotic driving forces
$\kappa^{-1}$	Debye length, m
$\kappa^{2}$	Debye–Hückel parameter, m${}^{-2}$
λ	relaxation time, s
ϕ	external electric potential, V
ψ	electric potential, V
$\Psi_{1}$	first normal stress coefficient, kgm${}^{-1}$s${}^{-2}$
ρ	fluid density, kgm${}^{-3}$
$\rho_{e}$	electric charge density, Cm${}^{-3}$
$\tau_{ii}$	normal stresses, kgm${}^{-1}$s${}^{-2}$
$\tau_{xy}$	shear stress, kgm${}^{-1}$s${}^{-2}$
ε	dielectric permittivity, CV${}^{-1}$m${}^{-1}$
ζ	zeta potential in the shear plane of the electric double layer, V
$\dot{\gamma }$	shear rate, s${}^{-1}$
$\bar{\kappa }$	ratio of the microchannel half-height to the Debye length
${\bar{\tau }}_{xx},{\bar{\tau }}_{yy}$	dimensionless normal stresses
${\bar{\tau }}_{xy}$	dimensionless shear stress

Mathematical
∇	codeformational time derivative
∘	corotational time derivative
⋄	configurational time derivative

Subscripts
sh	refers to Helmholtz-Smoluchowski
*x*	refers to the axial coordinate
0	indicates reference conditions
